# Establishment of a value assessment framework for orphan drugs in China: an application of the discrete choice experiment in multicriteria decision analysis

**DOI:** 10.3389/fphar.2025.1677627

**Published:** 2025-10-02

**Authors:** Zhihao Zhao, Xian Tang, Ming Hu

**Affiliations:** West China School of Pharmacy, Sichuan University, Chengdu, Sichuan, China

**Keywords:** orphan drugs, basic medical insurance access, value assessment framework, multicriteria decision analysis, discrete choice experiment

## Abstract

**Objectives:**

The medical security of orphan drugs faces difficulty in basic medical insurance access in China. Conventional cost-effectiveness analysis in the access process lacks broader value concerns and a value assessment framework is needed. This study aims to construct a multicriteria decision analysis value assessment framework for orphan drugs using the discrete choice experiment method from the perspective of basic medical insurance access in China.

**Methods:**

The attributes and levels of the framework were identified through literature and reports review. An unlabeled discrete choice experiment was employed to determine the relevance and relative importance weight of such attributes for decision-making. Questionnaire was designed based on D-efficient design. Survey was conducted anonymously using an online survey platform. A mixed logit model estimated the DCE responses.

**Results:**

Seven attributes (disease severity, unmet needs, drug efficacy, improvement in health-related quality of life, drug safety, quality of drug evidence, and annual treatment cost per patient reimbursed by basic medical insurance) were selected with three levels for each. It formed three parallel questionnaires, each containing 11 paired choice sets. A total of 84 respondents completed the study, and 69 questionnaires were valid. The results showed that six of the seven attributes were significant, except for ‘Unmet needs’. Among all attributes and levels, the respondents exhibited the highest WTP (567,900 RMB/year) for ‘significant improvement in usual activities’. Based on discrete choice model, the most important attributes measured by their relative importance weights are: improvement in health-related quality of life (23.44%), disease severity (18.65%) and annual treatment cost per patient reimbursed by basic medical insurance (17.34%). Different types of respondents and weighting methods may lead to slight variations in the results.

**Conclusion:**

Our study provides a new research perspective and methodological support for the value assessment of orphan drugs. When establishing a value assessment framework for orphan drugs in China, overall, the medical insurance access prioritized disease severity, and improvement in health-related quality of life. The application of discrete choice experiment proves to be a powerful tool for weighting criteria in healthcare multicriteria decision analysis framework and should be further explored for the value assessment of orphan drugs. Our findings offer a structured, evidence-based framework to support access and reimbursement decisions for orphan drugs.

## 1 Introduction

Rare diseases are characterized by low incidence rates and high disease burden (Ferreira, 2019). Despite the lack of a universal definition (Gong et al., 2016), they are generally categorized by the number of patients, incidence rate, and the severity of the disease (Ferreira, 2019; Richter et al., 2015). Although the prevalence of rare diseases is low, the accumulated number of affected patients is high. This results in a heavy disease burden, which reflected in high mortality rates, high disability rates (Ferreira, 2019).

Orphan medicinal products, also referred to as orphan drugs, are vital for diagnosing, preventing, and treating rare diseases (Joppi and Garattini, 2013). However, the medical security of orphan drugs still faces barriers including limited market accessibility, high costs exceeding patient affordability, and, especially, difficulty in basic medical insurance access. Generally, the scarce and low-quality evidence and high price of orphan drugs lead to unfavorable incremental cost-effectiveness ratio (ICER) and significant budget impact (Lasalvia et al., 2019), which are considered in the basic medical insurance access. Additionally, the traditional cost-effectiveness analyses (CEA) used in basic medical insurance access were unable to capture broader value concerns (McQueen et al., 2024) vital to orphan drugs. Based on these reasons, conventional health technology assessment (HTA), focusing on the measurement of value as captured by comparative effectiveness (Oliveira et al., 2019), is unsuitable for orphan drugs’ value assessment and access decisions. Establishing a value-based assessment mechanism specific to orphan drugs is therefore essential to support decision-making.

To address increasing healthcare demands under limited resources, health systems are shifting towards value-based reimbursement (Zhang M. et al., 2022). Accordingly, many HTA agencies have developed various value assessment frameworks to support the evaluation of new treatment interventions and optimize coverage decisions. Currently, there are three approaches commonly used to construct value assessment frameworks: deliberation, expanding beyond the traditional CEA, and multi-criteria decision analysis (MCDA) (Zhang M. et al., 2022; Zhang et al., 2024). But the former methods (deliberation and expanding CEA) suffer from a lack of transparency and difficulties in capturing a broader range of value elements (Zhang et al., 2024; Phelps et al., 2018; Angelis and Kanavos, 2017). MCDA, also called multiple criteria decision making (MCDM), refers to formal approaches which seek to take explicit account of multiple criteria in helping individuals or groups explore decisions that matter (Belton and Stewart, 2002). MCDA is widely applied in decision-making across fields such as the environment, energy (Hussain et al., 2025a; Rukhsar et al., 2025; Hussain et al., 2025b; Hussain et al., 2024a), and education (Hussain et al., 2025c), and it has developed advanced decision-making methodologies.Also, it is increasingly being adopted in health systems. It is worth noting that due to different objectives, the specific application methods of MCDA vary across different fields. Due to its unique advantages, MCDA has become an ideal tool for implementing value-oriented health decision-making (Nie et al., 2023). Many studies have adopted the MCDA method to construct the evaluation framework, as this approach stands out due to its flexibility, transparency, and the ability to incorporate the viewpoints of stakeholders (Blonda et al., 2021). This approach can address the shortcomings of the conventional HTA methods for orphan drugs, providing support to medical insurance decision-makers (Sussex et al., 2013; Palaska and Hutchings, 2015; Schey et al., 2017).

Specifically for orphan drugs, a 2021 study systematically summarized six main approaches to construct value assessment frameworks: no economic evaluation, standard economic evaluation, variable ICER thresholds, weighted QALYs, MCDA frameworks, and separate frameworks for ultra-rare diseases (Blonda et al., 2021). Their findings suggest that compared to other types of frameworks, MCDA frameworks are highly flexible, allow stakeholder perspectives to be integrated, and thus provide a promising direction for rare disease value assessment. Several studies further highlight the utility of MCDA in rare disease contexts. Iskrov et al. (2014) demonstrated that MCDA can improve fairness and rigor in orphan drug reimbursement decisions (Iskrov and Stefanov, 2014). García-Diego et al. (2024) applied MCDA to comprehensively evaluate gene therapy for hemophilia, capturing its additional value (García-Diego et al., 2024). Chen et al. (2024) developed an MCDA framework for orphan drugs tailored to the Chinese context (Chen et al., 2024).

Various methods, such as direct rating and choice experiments, were used to determine the weights of attributes in MCDA-based value assessment frameworks. However, the direct weighting methods, which are prevalent among MCDA weighting methods, rely heavily on subjective judgment. Discrete choice experiment (DCE) is a stated preference survey method that reveals individuals’ preferences and decision-making behaviors when faced with multiple alternatives (Tan et al., 2022; Hauber et al., 2016). DCE has become popular among MCDA methods (Tan et al., 2022) and it may result from its flexibility in permitting evaluation of conflicting preferences by MCDA (Byun et al., 2016). Therefore, there is a need to explore ways to optimize weighting methods within MCDA and DCE seems one of the most commonly used methods to conduct further research on the selection and optimization of weighting methods and other key aspects (Tan et al., 2022). Although DCE has seen some application in MCDA, its implementation in MCDA for orphan drugs still needs to be explored.

China has placed significant emphasis on rare diseases in recent policies (Ying et al., 2021). Since initiating national reimbursement drug negotiations based on HTA in 2018, China has expanded coverage for many orphan drugs in National Reimbursement Drug List (NRDL). However, many high-cost orphan drugs are still excluded and the current NRDL access procedures and evaluation methods for orphan drugs are the same as those for common drugs, failing to capture their unique value (Yuan and Wu, 2021). It still faces limitations in evaluating the value of orphan therapies through more domains and criteria beyond the current HTA framework. There is a consensus (Zhang B. et al., 2022) suggesting that MCDA should be used to assess the comprehensive clinical value of orphan drugs in China. Also, the previous study has used DCE methods to explore the societal preferences for orphan drugs in China (Tan et al., 2022). However, from the perspective of China’s basic medical insurance access, there is currently a lack of evidence regarding the combination of DCE and MCDA to establish value attributes and weights.

To fill this gap, our study aims to construct an MCDA framework from the perspective of basic medical insurance access, using the DCE method to assess the value of orphan drugs, specifically (1) determine the most relevant attributes in DCE (i.e., criteria in the MCDA framework): for decision-making; (2) prioritize these value attributes according to their relative importance based on the preferences stated.

## 2 Methods

### 2.1 Study design

DCE involves designing a series of hypothetical choice scenarios, where respondents are asked to choose between options with different combinations of attributes. Each of their choices reflects a trade-off between the attributes and levels of the different options and this allows for assessing their relative importance in decision making (Nie et al., 2023; Ryan and Farrar, 2000).

**FIGURE 1:**
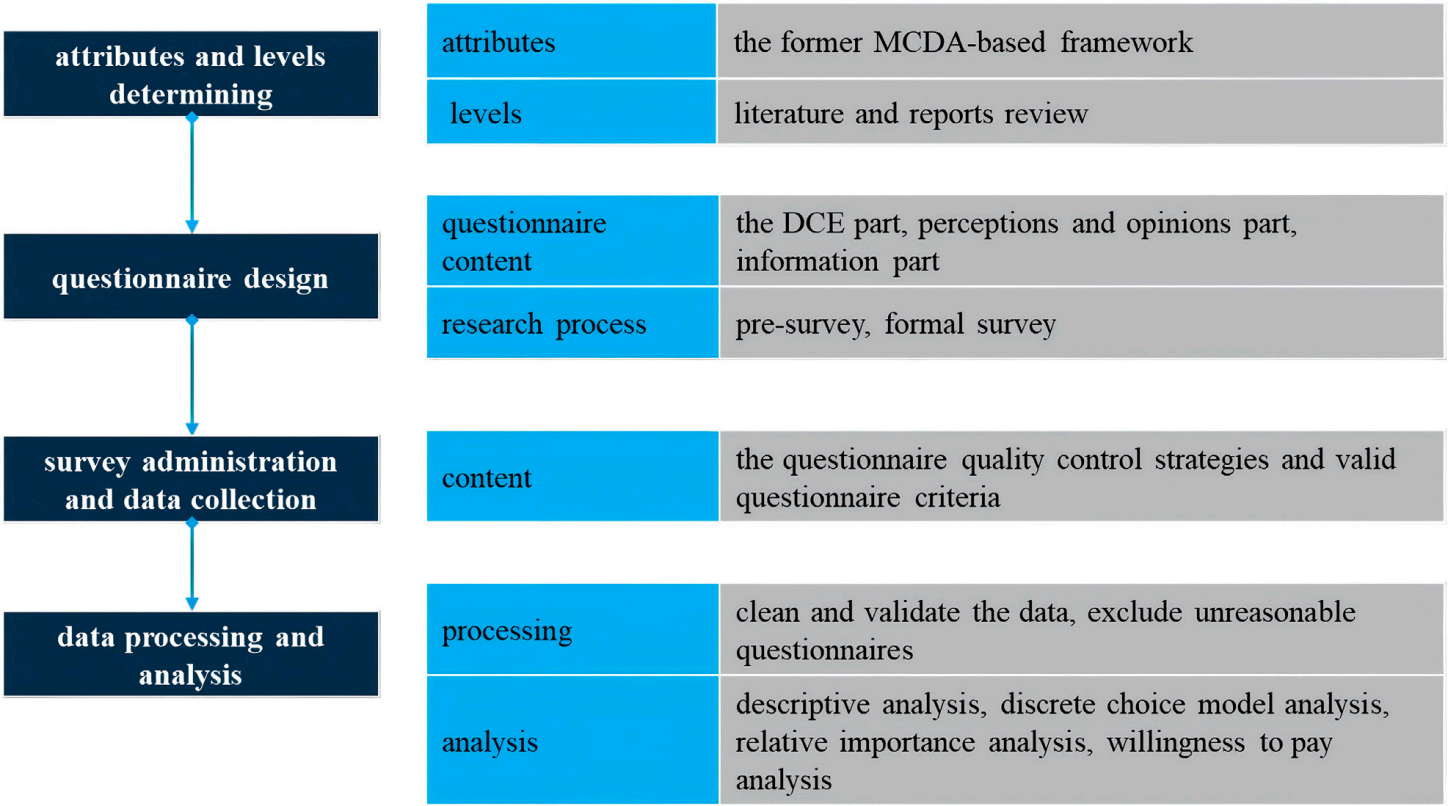
Flowchart of the study.

In 2011, the International Society for Pharmacoeconomics and Outcomes Research (ISPOR) developed a detailed checklist to assess the completeness and rigor of DCE studies (Bridges et al., 2011), and provided detailed guidance on experimental designs (Reed Johnson et al., 2013), data analysis methods (Hauber et al., 2016), and the accounting for preference heterogeneity (Vass et al., 2022) in DCE implementation. Following these checklist, our study was structured into four key steps: 1)attributes and levels determining, 2)experimental design, 3) survey administration and data collection, 4)statistical analyses. The flowchart of the study is shown in Figure 1.

### 2.2 Attributes and levels identification

The domains and attributes were developed by analyzing and integrating the former MCDA-based value assessment framework (Chen et al., 2024). The reason why this study selected the framework is that it is specifically constructed within the Chinese drug access context and serves as a comprehensive value assessment framework applicable to orphan drugs as a whole. Also, this former framework is developed based on EVIDEM, which is an collaborative-development MCDA framework designed to assess the value of interventions. The initial framework included a quantitative and a qualitative criteria part, which were divided into seven domains and 19 criteria. Due to sample size limitations and the potential cognitive burden on respondents, only the most important attributes and levels were selected (Wang et al., 2020). Criteria in the qualitative part were excluded from the final attributes because they mainly discussed compatibility or feasibility. Criteria in the qualitative part were removed, revised and combined through literature review and research team discussion. The process for detailed consideration of the attributes is documented in the Supplementary Material.

Attribute levels were derived from a literature review, some reports and guidelines of rare diseases and orphan drugs in China, such as *China rare disease drug accessibility report (2019)* (IQVIA, 2019), *the Guidelines for the Diagnosis and Treatment of Rare Diseases (2019)* (National Health Commission of the People’s Republic of China, 2019), clinical evidence, and global orphan drug preference studies. Level selection criteria ensured: 1) covering most therapeutic methods approved or under consideration for approval in China, 2) ensuring sufficient differentiation and exclusivity between each level, and 3) balancing the total number of levels. The disease-related domain’s levels were established by systematic analysis of classifications, onset ages, and severity of rare disease listed in the first and second ‘List of Rare Diseases’ in China; drug/treatment-related levels were defined based on the extent to which rare disease can be treated (curative, controllable, or symptomatic/supportive treatment) and the efficacy of controlling treatment (significant improvement, partial improvement, or stable control); cost-related levels were determined by an analysis of the inclusion of orphan drugs in the NRDL, drug costs under different medical insurance types, and China’s basic medical insurance policies and current status.

Finally, this DCE includes seven attributes, with three levels for each attribute (Table 1). The specific definitions and explanations of each attribute and level, along with the rationale for each level, can be found in Supplementary Material.

**TABLE 1:** Attributes and levels.

Domains	Attributes	Levels
disease-related	Disease severity	Low
		Moderate
		High
drug/treatment-related	Unmet needs	Mature treatments available with good clinical outcomes
		Controllable treatments available to manage disease progression
		No specific treatment available, only symptomatic/supportive treatment
	Drug efficacy	Stabilizes disease
		Partially improves or alleviates
		Significantly improves or alleviates
	Improvement in health-related quality of life	No improvement in usual activity
		Partial improvement in usual activity
		Significant improvement in usual activity
	Drug safety	May cause severe adverse reactions
		May cause moderate adverse reactions
		No or mild adverse reactions
	Quality of drug evidence	Low
		Moderate
		High
cost-related	Annual treatment cost per patient reimbursed by basic medical insurance	500,000 RMB
		200,000 RMB
		80,000 RMB

### 2.3 Experimental design

#### 2.3.1 DCE choice set design

The full factorial design would generate 2187 possible alternatives (3^7^ = 2187) based on seven attributes and three levels for each. Given the practical constraints of the research, a fractional factorial design was employed to select representative experimental programs from the many possible combinations for DCE construction. A D-efficient design was used to obtain 30 paired choice sets using Stata 18. To reduce the burden of respondents and to ensure data quality, these were equally divided into three blocks.

**FIGURE 2:**
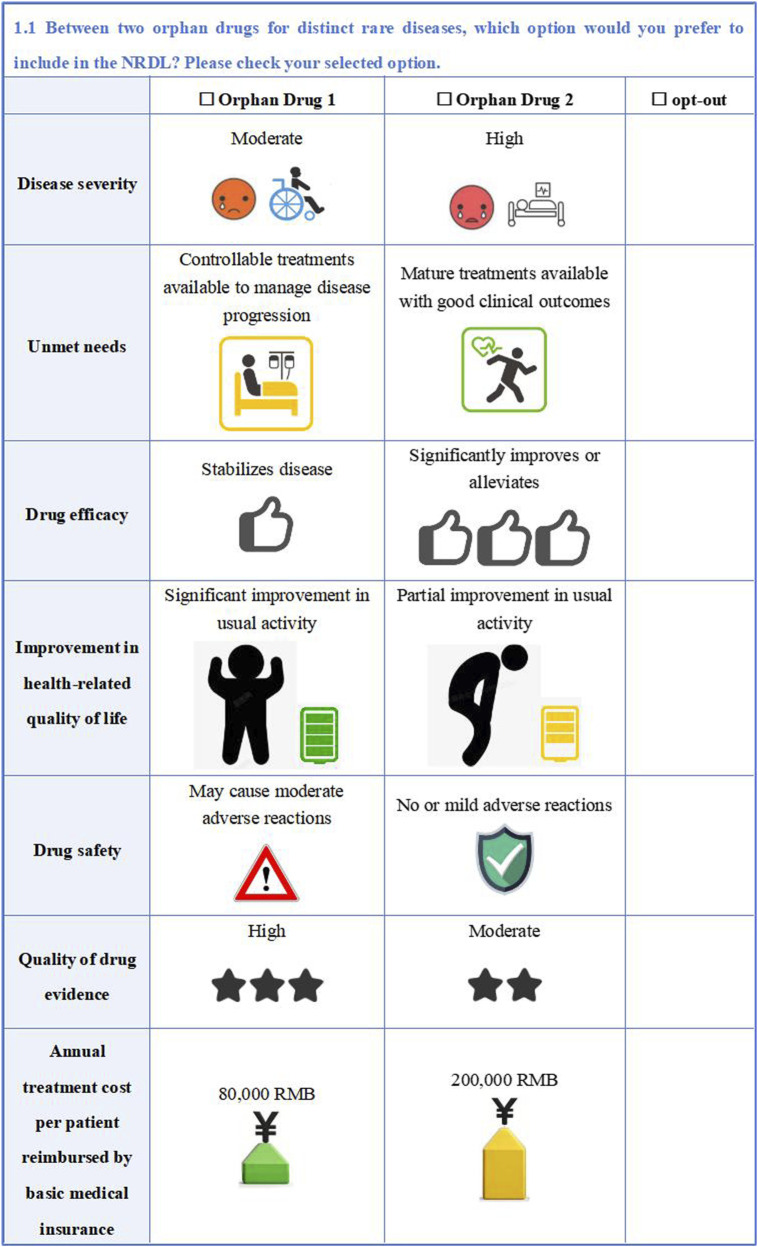
An example of DCE questionnaire.

The study employed an unlabeled design for the DCE with alternatives generically labeled ‘Orphan Drug 1’ and ‘Orphan Drug 2’ to avoid comparative superiority between different orphan drug alternatives and focus participant evaluation on the specific drug value attributes. To better align with real-world selection situations, an ‘opt-out option’ was added to each choice set. For quality control, we added a set of repeated choice sets to each questionnaire as a basis for consistency validation to ensure the internal consistency. Hence, each respondent was required to complete 11 DCE choices. An example of DCE questionnaire is shown in Figure 2.

#### 2.3.2 Questionnaire design

The questionnaire included three parts: the DCE questionnaire, perceptions and opinions on each attribute of orphan drugs, and socio-demographic information.

The questionnaire began with a study overview and instructions. In the ‘DCE questionnaire on the assessment of the value of orphan drugs’ part, respondents were given detailed attribute and level explanations and an example choice task. After completing the choice sets, respondents evaluated task clarify and difficulty.

In the ‘perceptions and opinions on each attribute of orphan drugs’ part, Simple Multi-Attribute Rating Technique (SMART), which was a direct scoring method, was used to assess the relative importance of the attributes of orphan drugs. Respondents are asked to: 1)rank the importance of each attribute; 2) assign scores (0–100) to each attributes based on relative importance, and 3) provide additional relevant insights or recommendations.

The final part collected information including gender, age, education level, field of work, place of residence, years of experience, current workplace.

After completing the initial questionnaire design, a small-scale pre-survey was conducted. Based on the feedback from the respondents, the formal research questionnaire was optimized and adjusted.

### 2.4 Survey administration and data collection

This study administered surveys via the online platform, distributing three randomized questionnaire versions to respondents.

The inclusion criteria for respondents were: (1) invited basic medical insurance experts, including administrators or managers from different levels of government or hospital-based medical insurance departments; (2) invited health economics experts who were scholars specializing in health economics and pharmacoeconomics from universities and research institutions across China; (3) informed consent.

The survey was conducted from January 2025 to April 2025. During the questionnaire survey, quality control strategies were implemented to ensure the reliability of the data, specifically including: (1) electronic data collection with immutable entry upon submission; (2) researchers provided timely support; (3) regular reviews of the collected data.

Valid questionnaire criteria were: (1) Completion time ≥8 min; (2) Passing the DCE internal consistency test; (3) If meeting (1) but failing (2), responses were still deemed valid provided that attribute importance rankings in the SMART part logically aligned with corresponding importance scores.

### 2.5 Statistical analysis

Microsoft Excel 2019^®^ was used to clean and validate the collected data and exclude unreasonable questionnaires. The statistical analysis included descriptive analysis, discrete choice model analysis, relative importance analysis, and willingness to pay analysis. All data analyses were done by Stata 18.

#### 2.5.1 Descriptive statistical analysis

Descriptive statistics were used for each variable, with frequency and percentage for categorical variables.

#### 2.5.2 Discrete choice model analysis

Based on random utility theory, the study conducted a discrete choice model to reveal the overall preferences of respondents and their heterogeneity. The model assumes that respondent *i* chooses option *j* because the utility provided by option *j* is higher than that of all other options (Mangham et al., 2009). Utility was calculated using the following formula:

$$U_{ij}=V_{ij}\left(X_{ij}\right)+\varepsilon_{ij}=\beta X_{ij}+\varepsilon_{ij}=\beta_{0}+\beta_{1}{\text{Severity}}_{1}+\beta_{2}{\text{Severity}}_{2}+\beta_{3}{U\text{nmet}}_{1}+\beta_{4}{U\text{nmet}}_{2}+\beta_{5}{\text{Efficacy}}_{1}+\beta_{6}{\text{Efficacy}}_{2}+\beta_{7}{\text{HRQoL}}_{1}+\beta_{8}{\text{HRQoL}}_{2}+\beta_{9}{\text{Safety}}_{1}+\beta_{10}{\text{Safety}}_{2}+\beta_{11}{\text{Evidence}}_{1}+\beta_{12}{\text{Evidence}}_{2}+\beta_{13}C\text{ost}+\varepsilon_{ij}$$

Note: where, $U_{ij}$ represents the utility value obtained by respondent *i* after choosing a particular orphan drug option *j*. $X_{ij}$ is the independent variable related to the attribute, $V_{ij}\left(X_{ij}\right)$ represents the utility directly observable when choosing the orphan drug option *j*. The random term $\varepsilon_{ij}$ represents the unobservable error, and the program-specific constant $\beta_{0}$ represents the coefficient of the opt-out option (ASC for opt-out). The $\beta_{1}$ - $\beta_{13}$ represents the attribute level coefficient, which indicates the preference weight and relative importance of the respondents toward the attribute levels.

The DCE data were analyzed using a mixed logit model. The selection of the model is judged by the Akaike information criterion (AIC) and the Bayesian information criterion (BIC). The significance level for statistical analysis was set at 0.05.

#### 2.5.3 Relative importance analysis

The aim of the relative importance analysis was to weigh and prioritize the attributes. The study conducted both the direct and indirect weighing method.

##### 2.5.3.1 Relative importance analysis based on discrete choice model

Relative importance (RI) refers to the magnitude of an attribute’s preference relative to the other attributes, reflecting the decision maker’s overall preference at the attribute level (Lancsar et al., 2007). In this study, the relative importance of attributes is calculated based on the construction of the discrete choice model to compute the weight of each attribute’s importance (Yang et al., 2025).

The specific formula for calculating the relative importance of attribute one is:

$$RI=\frac{\beta_{1\max}-\beta_{1\min}}{\sum \left(\beta_{cmax}-\beta_{cmin}\right)}$$

Note: where, $\beta_{\max}\text{ and }\beta_{\min}$ represent the maximum and minimum regression coefficients, respectively; c = 1,2,3,....

##### 2.5.3.2 Relative importance analysis based on SMART

According to the second part of the questionnaire, the distribution of attribute rankings was analyzed to calculate the average and overall rankings based on the SMART method. Individual-level attribute weights were derived by normalizing the importance scores provided by each respondent, and the average weights across all respondents were then computed to reflect the overall importance scores.

#### 2.5.4 Willingness to pay analysis

Willingness to Pay (WTP) represented the marginal utility respondents were willing to pay for a particular change in attribute level and that represented the relative importance of each attribute level (Tan et al., 2022). Among the seven attributes in this study, the annual treatment cost per patient reimbursed by basic medical insurance was treated as a continuous variable to calculate WTP for each attribute level of orphan drugs using the following formula:

$$\text{WTP}=-\frac{\beta_{\text{attribute}\;\text{level}}}{\beta_{\text{cost}}}$$

Note: where, $\beta_{\text{attribute}\;\text{level}}$ represents the preference weight coefficients for each attribute level and $\beta_{\text{cost}}$ represents the preference weight coefficients for the annual treatment cost per patient reimbursed by basic medical insurance.

## 3 Results

### 3.1 Respondent characteristics and questionnaire response

A total of 84 decision-makers participated in filling out the questionnaires, and 69 (83.3%) valid questionnaires were included, with 37 health economic experts and 32 basic medical insurance experts. Among all included respondents, 60.87% were male, with the majority (75.36%) aged 30–49 years. Half of them (50.72%) held a doctoral degree, and all were health economics experts. Most of them had been in this field for 10–19 years (52.17%) (Table 2).

**TABLE 2:** Basic characteristics of the study sample.

Characteristics	All *(n* = 69)		Health economics experts *(n* = 37)		Basic medical insurance experts *(n* = 32)	
	*n*	(%)	*n*	(%)	*n*	(%)
Gender						
Male	42	60.87	22	59.46	20	62.50
Female	27	39.13	15	40.54	12	37.50
Age						
20–29 years	1	1.45	0	0.00	1	3.13
30–39 years	31	44.93	13	35.14	18	56.25
40–49 years	21	30.43	16	43.24	5	15.63
50–59 years	14	20.29	6	16.22	8	25.00
60–69 years	2	2.90	2	5.41	0	0.00
Education level						
Bachelor	16	23.19	0	0.00	16	50.00
Master	18	26.09	2	5.41	16	50.00
Doctor	35	50.72	35	94.59	0	0.00
Years of experience						
0–9 years	17	24.64	6	16.22	11	34.38
10–19 years	36	52.17	21	56.76	15	46.88
20–29 years	9	13.04	6	16.22	3	9.38
30 years and above	7	10.14	4	10.81	3	9.38

### 3.2 Preferences based on mixed logit modeling

#### 3.2.1 Preferences of attributes

Table 3 shows the results of the mixed logit model analysis. Overall, all attribute levels were statistically significant except for the two levels of the attribute ‘Unmet needs’.

**TABLE 3:** Preferences results based on mixed logit modeling.

Attributes and levels	All *(n* = 69)		Health economics experts *(n* = 37)		Basic medical insurance experts *(n* = 32)	
	β	SE	β	SE	β	SE
out-put	2.454***	0.506	5.363***	1.247	1.939*	0.947
Annual treatment cost per patient reimbursed by basic medical insurance	−0.039***	0.006	−0.067***	0.017	−0.053***	0.016
Disease severity (ref. Low)						
Moderate	1.509***	0.303	2.936***	0.731	1.428**	0.544
High	1.753***	0.313	3.877***	0.851	1.296*	0.574
Unmet needs (ref. Mature treatments available with good clinical outcomes)						
Controllable treatments available to manage disease progression	−0.072	0.210	0.614	0.345	−0.577	0.361
No specific treatment available, only symptomatic/supportive treatment	0.361	0.281	1.917***	0.543	−1.017	0.523
Drug efficacy (ref. Stabilizes disease)						
Partially improves or alleviates	0.531*	0.245	0.888*	0.432	0.590	0.450
Significantly improves or alleviates	1.120***	0.227	2.354**	0.697	1.054*	0.429
Improvement in health-related quality of life (ref. No improvement in usual activity)						
Partial improvement in usual activity	1.610***	0.309	2.359***	0.561	2.047**	0.690
Significant improvement in usual activity	2.204***	0.330	3.995***	1.042	2.433***	0.666
Drug safety (ref. May cause severe adverse reactions)						
May cause moderate adverse reactions	1.037***	0.280	1.178*	0.462	1.512**	0.562
No or mild adverse reactions	1.231***	0.279	1.011*	0.435	2.355**	0.774
Quality of drug evidence (ref. Low)						
Moderate	1.028***	0.240	0.906*	0.379	1.594***	0.462
High	0.676**	0.256	1.061*	0.521	0.808	0.417
Log likelihood	−575.88767		−281.83149		−254.76378	
AIC	1203.775		615.663		561.5276	
BIC	1350.293		745.978		688.0678	

*: P ≤ 0.05; **: P ≤ 0.01; ***: P ≤ 0.001; AIC: akaike information criterion; BIC: bayesian information criterion; β, coefficient; SE, standard error.

The overall results of all the respondents and the subgroup results (basic medical insurance experts and health economics experts) show both consistencies and differences across various attributes. The preference coefficient for the ‘Annual treatment cost per patient reimbursed by basic medical insurance’ was significantly negative for all decision-makers (*β* = −0.039***) and subgroups (basic medical insurance experts: *β* = −0.053***; health economics experts: *β* = −0.067***), indicating that the higher the cost, the lower possibility that this drug included into the NRDL. The preference coefficient for ‘Improvement in health-related quality of life-Significant improvement in usual activity’ was the largest overall (*β* = 2.204***), and the results were similar for both subgroups. However, there were some differences between the subgroups. In terms of ‘Unmet needs’, health economics experts were nearly 2 times more likely to select ‘No specific treatment available’ orphan drugs for NRDL access compared to drug with ‘Mature treatments available’ (*β* = 1.917***), while the basic medical insurance experts showed a non-significant trend in preference (*β* = −1.017).

#### 3.2.2 Willingness-to-pay

The willingness to pay analysis results were shown in Table 4. Among all attributes levels, the decision-makers exhibited the highest WTP for ‘significant improvement in usual activities’ (*β* = 56.79***). Additionally, decision-makers expressed a high WTP for levels of the severity of the disease. Specifically, compared to orphan drugs for low severe diseases, they preferred to pay 388,900 RMB per year for orphan drugs that treat diseases of moderate severity, and 451,900 RMB per year more for medications that treat diseases of high severity.

**TABLE 4:** Results of willingness-to-pay analysis (10,000 RMB/year).

Attributes and levels	All *(n* = 69)		Health economics experts *(n* = 37)		Basic medical insurance experts *(n* = 32)	
	β	SE	β	SE	β	SE
Disease severity (ref. Low)						
Moderate	38.89***	8.13	44.12***	8.38	27.16**	9.14
High	45.19***	8.09	58.26***	9.24	24.63**	8.51
Unmet needs (ref. Mature treatments available with good clinical outcomes)						
Controllable treatments available to manage disease progression	−1.87	5.38	9.22	5.50	−10.98	7.47
No specific treatment available, only symptomatic/supportive treatment	9.30	7.47	28.80**	9.68	−19.34*	8.27
Drug efficacy (ref. Stabilizes disease)						
Partially improves or alleviates	13.68*	6.23	13.35*	5.86	11.23	8.18
Significantly improves or alleviates	28.87***	6.13	35.38***	7.19	20.04**	7.12
Improvement in health-related quality of life (ref. No improvement in usual activity)						
Partial improvement in usual activity	41.49***	8.11	35.45***	7.63	38.91***	9.63
Significant improvement in usual activity	56.79***	9.34	60.03***	9.93	46.25***	10.97
Drug safety (ref. May cause severe adverse reactions)						
May cause moderate adverse reactions	26.73***	6.82	17.70**	6.47	28.74***	8.47
No or mild adverse reactions	31.72***	7.23	15.20**	5.74	44.77***	9.95
Quality of drug evidence (ref. Low)						
Moderate	26.50***	6.50	13.62*	5.71	30.30***	9.09
High	17.42**	6.62	15.94*	6.80	15.36	8.50

*: P ≤ 0.05; **: P ≤ 0.01; ***: P ≤ 0.001; β, coefficient; SE, standard error.

In terms of subgroups, regarding disease severity, compared to moderate severity, health economics experts were more concerned about high severity diseases, with their willingness to pay a 32% premium, while basic medical insurance experts did not show a higher preference for high disease severity. For ‘Unmet need’, health economics experts showed a high WTP (288,800 RMB/year **) for diseases with ‘No specific treatment available’ while basic medical insurance experts were significantly opposed to the inclusion (−193,400 RMB/year *). Overall, basic medical insurance experts demonstrated a higher WTP for better drug safety and evidence quality, while health economics experts showed a relatively higher WTP for other attributes.

### 3.3 Weighting and prioritization of the attributes

#### 3.3.1 Relative importance based on discrete choice modelling

The results were shown in Table 5. For all decision-makers, ‘Improvement in health-related quality of life’ was the most important attribute, with a relative importance weight of 23.44%. This was followed by ‘Disease severity’ (18.65%) and ‘Annual treatment cost per patient reimbursed by basic medical insurance’ (17.34%). ‘Quality of drug evidence’ (10.94%) and ‘Unmet need’ (4.61%) had relatively lower weights and ranks.

**TABLE 5:** Results of relative importance analysis based on DCE.

Attributes	RI		
	All *(n* = 69)	Health economics experts *(n* = 37)	Basic medical insurance experts *(n* = 32)
Improvement in health-related quality of life	23.44%	23.26%	20.12%
Disease severity	18.65%	22.57%	11.81%
Annual treatment cost per patient reimbursed by basic medical insurance	17.34%	16.27%	18.27%
Drug safety	13.10%	6.86%	19.48%
Drug efficacy	11.92%	13.71%	8.72%
Quality of drug evidence	10.94%	6.18%	13.18%
Unmet needs	4.61%	11.16%	8.41%

It is evident that ‘Improvement in health-related quality of life’ was the highest-weighted attribute in all groups. ‘Disease severity’ (22.57%) was the second most important attribute for the health economics experts group. But the second most important attribute for the basic medical insurance experts was ‘Drug safety’, which was 2.84 times more important than the weight by the health economics experts.

#### 3.3.2 Relative importance based on SMART

It was shown that the order of the average ranking was different from the order of the scoring results, but the top three attributes with higher weights were the same. In terms of average ranking, ‘Disease severity’ had the highest ranking (2.75), followed by ‘Drug efficacy’ (2.80) and ‘Improvement in health-related quality of life’ (3.54). In terms of average scoring, ‘Drug efficacy’ had the highest weight of 16.22%, followed by ‘Improvement in health-related quality of life’ (15.94%) and ‘Disease severity’ (15.18%). The attributes with the lowest weights were ‘Quality of drug evidence’ for both ranking and scoring (Table 6).

**TABLE 6:** Results of relative importance analysis based on SMART.

Attributes	Ranking		Scoring	
	Average	Overall ranking	Average	Overall ranking
Disease severity	2.75	1	15.18%	3
Unmet needs	3.65	4	13.29%	5
Drug efficacy	2.80	2	16.22%	1
Improvement in health-related quality of life	3.54	3	15.94%	2
Drug safety	4.61	5	13.79%	4
Quality of drug evidence	5.46	7	12.40%	7
Annual treatment cost per patient reimbursed by basic medical insurance	5.19	6	13.18%	6

## 4 Discussion

### 4.1 Discussion of key findings

From the relative importance of the attributes derived from the DCE method, all respondents assigned a higher weight to ‘Improvement in health-related quality of life’, indicating that this attribute holds a central position in the value assessment of orphan drugs. Health-related quality of life (HRQoL) indicators (e.g., ability to usual activities, emotional state, social participation) directly reflect the impact of treatment on patients’ actual lives, integrating multidimensional values that traditional clinical endpoints (e.g., survival rate, laboratory indicators) may fail to capture. Improving HRQoL (as measured by QALY) has now become a core indicator in basic medical insurance assessments, which also contributes to long-term health economic considerations. Additionally, all respondents assigned a high weight to ‘Disease severity’ reflecting the ethical principle of ‘prioritizing urgent needs’ (Grover et al., 2020), which tends to allocate limited resources to populations with the highest disease burden. Furthermore, the moral responsibility towards vulnerable groups in our societal values contributes to public support for prioritizing extreme health conditions (Tan et al., 2022). However, we have also noticed that, compared to health economics experts, basic medical insurance experts tend to prioritize ‘Drug safety’ over ‘Disease severity’. This may reflect the fact that basic medical insurance experts first consider public safety, social stability, and fund stability, and need to guard against the risk of fund depletion caused by adverse reactions. On the other hand, when conducting NRDL access calculations, health economics experts may lack long-term safety evidence for new drugs, and are more concerned with quality of life and long-term benefits, so they place greater emphasis on ‘Disease severity’. Further research is needed to determine the causes of this discover.

The current sample selection and characteristics may influence the results. Regarding education level, 94.59% of health economics experts hold doctoral degrees, whereas basic medical insurance experts have master’s degrees (50%) and bachelor’s degrees (50%) as their highest qualifications, potentially leading to perspective biases. Medical insurance experts are more likely to come from practice-oriented backgrounds, while health economics experts tend to focus on theoretical research, methodological innovation, and long-term health outcomes. Similarly, in terms of age and work experience, the experts in this study are predominantly middle-aged and older with rich experience. This may result in a lack of innovative perspectives from younger generations, making them more conservative in accepting new value elements. Likewise, the absence of physician and patient samples in this study may lead the framework to ignore other crucial value dimensions in the treatment process.

### 4.2 Discussion of comparative study

The attributes of orphan drugs in this study were based on the MCDA framework established in a previous study (Chen et al., 2024), and the weights of the criteria in the original framework were based on a two-step percentile distribution method obtained from 13 stakeholders (including 3 health economics experts, 3 health insurance decision-makers, 3 clinicians, 2 clinical pharmacists, and 2 patients). Compared with previous study, the relative importance of ‘Disease severity’ was higher in both studies, which can be recognized as the most critical factor in assessing the value of orphan drugs, and is therefore an important factor to consider when NRDL access. However, the discrete choice model in this study yielded the highest weighting for ‘Improvement in health-related quality of life’ compared to the previous framework, whereas the weighting for ‘Comparative patient-perceived health/patient-reported outcomes’ was lower than that for drug benefits and effectiveness. This may reflect a paradigm shift in healthcare assessment from traditional clinical indicators to patient-centered outcomes. The reasons for this change may stem from policy drivers, such as the progressive requirement by medical insurance decision-makers to consider patient-reported outcomes (PROs) and HRQoL. Also, due to the differences in the definition and scope of the attributes, the ‘Improvement in health-related quality of life’ attribute in our study includes the overall impact of the drug on the patients’ health, whereas the ‘Comparative patient-perceived health/patient-reported outcomes’ in the original framework may be limited to symptomatic outcomes and did not adequately cover functional recovery or social participation. On the other hand, in this study, the results of the weighting of ‘Unmet needs’ ranked lower in importance, possibly due to respondents’ preference for easy-to-understand attributes such as ‘Drug efficacy’ and ‘Drug safety’. ‘Unmet need’ involves hypothetical situations and counterfactual thinking and that will cause higher cognitive load and finally lead to lower decision weight. Moreover, this study compared the weights of attributes in this study with those of the orphan drugs’ value assessment framework derived from other studies in China (Yuan and Wu, 2021; Hu et al., 2018). The results showed that there were some differences in the setting of the criteria, and some of the weighting results showed consistency. For instance, the indicators related to therapeutic efficacy were generally regarded as one of the most critical indicators, followed closely by disease severity and drug safety, which were also given higher importance. The differences in the results of different studies not only stem from the heterogeneity of the research objectives and methodologies, but also reflect the lack of a unified and standardized assessment system in the field of drug value assessment for rare diseases.

Also, this study use the SMART method to calculate the relative importance of each attribute. The ranking results derived from the SMART and DCE was inconsistent. For the relative importance for ‘Drug efficacy’, the SMART method produced higher weights compared to DCE. The reason maybe that the SMART method assigns weights through direct scoring (Dwanoko et al., 2018), and efficacy, due to its intuitive clinical significance, is easily overestimated. In contrast, the DCE method can simulate real-life choice scenarios (Wang et al., 2021). When efficacy is bundled with other high-cost or high-risk attributes (e.g., severe side effects), its priority in actual choices may be diluted. The relative importance of ‘Annual treatment cost per patient reimbursed by basic medical insurance’ also varied between the two methods. The SMART method assigned lower importance to this attribute, indicating that respondents may prefer orphan drugs with significant efficacy that greatly improve patients’ quality of life, even if higher treatment costs. In the DCE, since this attribute is a continuous variable, the values between levels may partially influence the weight calculation, leading to differences in the final weight distribution. Overall, the two methods have significant differences in theoretical foundations and operational processes. The SMART method offers simplicity and ease of use but may not capture subtle differences in preferences. While, the DCE method, although more complex, is better at accurately reflecting respondents’ preferences and provides richer information (Whichello et al., 2023).

### 4.3 Strengths, significance and challenges of the framework

This study established the relative importance of different criteria in MCDA framework for orphan drugs using the DCE method from the perspective of basic medical insurance in China. Our study provides a new research perspective and methodological support for the value assessment of drugs for rare diseases by integrating the strengths of DCE and MCDA. By comparing the weight and prioritization of two different types of respondents, namely, health economics experts and basic medical insurance experts, the framework reveals the core elements of the value assessment of orphan drugs and the heterogeneous concerns of different groups, providing important insights for decision-makers to balance the interests of multiple parties. In health insurance reimbursement or financing decisions, the MCDA method can enhance the transparency of decision-making and value judgments clearer, improving the consistency and repeatability of decisions (Baltussen et al., 2019). When weighting of criteria of MCDA, DCE helps researchers gain a deeper understanding of the individual choice process, revealing how different factors influence decision outcomes. Such insights assist in better understanding individual behaviors and decision-making, thereby providing strong support for decisions. The application of DCE proves to be a powerful tool for weighting criteria in healthcare multicriteria decision analysis framework.

At present, the mechanism of NRDL inclusion in China has established a systematic process of ‘preparation–application–expert review–negotiation/bidding’. The purpose of constructing an MCDA framework is to support decision-making. Therefore, the framework developed in this study can serve as supplementary material during the expert review phase and the final negotiation/bidding phase, helping to clarify the key value elements of the specific orphan drugs and better demonstrate their advantages for NRDL access.

The core dilemma in orphan drugs access lies in the trade-off between affordability for the healthcare system and the incentives for pharmaceutical innovation. Price negotiations can enhance drug accessibility by lowering costs, but may discourage companies from investing in orphan drug research and development. Conversely, inadequate cost control could jeopardize the sustainable operation of basic medical insurance funds. This tension is pronounced in orphan drug access. The value assessment framework of orphan drugs offers a structured solution: affordability considerations are incorporated through the cost-related attributes, while innovation-related social value is reflected in attributes such as disease severity, unmet need, drug efficacy and improvement in health-related quality of life. It enables decision-makers to weigh trade-offs within a more transparent framework.

However, the application of this value framework also faces challenges. First, rare diseases are characterized by small patient populations and uncertain long-term outcomes, often lacking HRQoL or long-term survival evidence. Second, China currently lacks national-level guidelines and toolkits for MCDA and DCE, potentially leading to implementation capacity gaps in practice. In addition, due to the flexibility of the framework, issues such as stakeholder representation and the determination of weights for specific orphan drugs may spark controversy.

### 4.4 Future study directions

Further research could validate and refine the value assessment framework of orphan drugs based on DCE and apply this framework to real-world decision-making contexts, providing practical evidence and data support for the optimization of the framework. At the same time, we recommended to explore advanced weighting methodologies and data analytics techniques to make the results more scientific and precise.

Also, in other fields, studies have proposed advanced algorithm-based MCDM approaches, aimed at more precisely handling uncertainty and complexity (Hussain et al., 2024b; Zhao et al., 2024; Ullah et al., 2025). Although this study primarily focuses on capturing stakeholders’ value preferences regarding orphan drug access, the above algorithmic approaches can provide new insights by introducing more rigorous mathematical modeling to enhance the robustness of conclusions.

### 4.5 Limitations

The limitations of this study are as follows. First, the questionnaire survey was conducted online via a web-based platform, rather than the traditional face-to-face method. Face-to-face interviews or surveys allow researchers to comprehensively capture respondents’ feedback and provide immediate assistance.

Second, the relatively small sample size and the current sample selection may affect the generalizability and accuracy of the findings. Future studies could consider expanding the sample scope to include more geographic regions and types of stakeholders, ensuring a balanced representation of respondents.

Third, our study does not cover specific disease types or conditions. Although the study provides useful information and conclusions, it would benefit from the inclusion of more real-world data and pharmacoeconomic evidence to further validate and refine the assessment framework.

Finally, sensitivity analysis was not performed in this study. Although sensitivity analysis is crucial for examining uncertainty in MCDA, few studies applying an MCDA framework using the DCE method in the field of HTA have conducted sensitivity analyses. This is likely because the primary objective of these studies was to construct the value assessment framework and its application remains limited to pilot or exploratory stages.

## 5 Conclusion

This study explored and compared the results of weighting MCDA criteria for orphan drugs by decision-making experts from basic medical insurance and health economics backgrounds using different methods, such as DCE and SMART. The results showed, from the perspective of basic medical insurance access, different types of decision experts and weighting methods may lead to slight variations in the results. However, disease severity and improvement in health-related quality of life are generally the two most important attributes influencing NRDL inclusion for orphan drugs. The application of the MCDA framework using the DCE method should be further explored for the value assessment of orphan drugs. The current framework still has some limitations in terms of sample selection, applicability, and sensitivity analysis. Future research could explore advanced weighting methodologies and data analytics techniques.

## Data Availability

The raw data supporting the conclusions of this article will be made available by the authors, without undue reservation.

## Abbreviations

MCDA, Multi-criteria Decision Analysis; NRDL, National Reimbursement Drug List; DCE, Discrete choice experiment; SMART, Simple Multi-Attribute Rating Technique; WTP, Willingness to pay; HRQoL, Health-related quality of life.
