# Supplementary material for: Establishment of a value assessment framework for orphan drugs in China: an application of the discrete choice experiment in multicriteria decision analysis
Source: Front Pharmacol. 2025 Oct 2;16:1677627. doi: 10.3389/fphar.2025.1677627 (PMC12527902; doi:10.3389/fphar.2025.1677627)
Supplement: Supplementary file 1 [file Supplementaryfile1.docx]

Supplementary material

**1 The modification, deletion, or supplementation of the former criteria**

(1) The former research results indicated that the criteria ‘**Comparative cost consequences–other medical costs’** and ‘**Comparative cost consequences–non-medical costs’** were the least important under both weighting methods. Based on the literature review, other related costs, except for direct medical costs, were generally not included in the limited DCE attribute settings. **Therefore, these two criteria were removed.**

(2) Since both ‘**Comparative effectiveness’** and ‘**Type of benefit of drugs’** describe the drug’s performance in terms of efficacy, but from different perspectives, merging these two attributes can avoid redundancy and reduce the number of attributes in the DCE. This will make the experimental design more compact and concise, thus **they were combined into ‘Drug efficacy’.**

(3) ‘**Quality of evidence**’ is a crucial consideration in both medical insurance access evaluation process and value assessments. High-quality evidence helps assess the cost-effectiveness of medications and ensures the effective use of funds. Since both ‘**Quality of evidence**’ and ‘**Expert consensus / clinical practice guidelines’** pertain to drug evidence and are similar in nature, expert consensus and clinical guidelines are usually based on existing best evidence, including results from randomized controlled trials (RCTs), systematic reviews, and meta-analyses. These two are essentially comprehensive interpretations of the available evidence. Therefore, they were combined into a single attribute **‘Quality of drug evidence’.**

(4) For the qualitative criterion **‘Affordability of medical insurance funds’,** based on former literature research, attributes such as ‘Medical insurance fund financing level’ and ‘Medical insurance payment costs’ frequently appeared in the value attributes of orphan drugs. Since the affordability of the medical insurance fund is crucial when making decisions on the inclusion of drugs in the NRDL, and stakeholders in previous research suggested incorporating it as a quantitative criterion, it was included. The cost of drug treatment (annual cost) is one of the key factors in determining whether a drug is included in the list, directly impacting the expenditure of the medical insurance fund. **‘Affordability of medical insurance funds’** determines whether the drug can be widely used and also reflects the financial health of the entire medical insurance system. **Thus, ‘Affordability of medical insurance funds’ and ‘Drug treatment cost (Annual Cost)’ were combined into the attribute ‘Annual treatment cost per patient reimbursed by basic medical insurance’**, reflecting the economic burden of treatment options on the medical insurance fund and helping understand the fund's capacity to bear costs at different levels, thus providing more comprehensive information for decision-making in practical scenarios.

(5) The remaining qualitative criteria mainly discuss their compatibility or feasibility, providing decision-makers with information to make the final reimbursement decision based on social conditions. **Therefore, these were not included in the DCE design.**

**2 The definitions and explanations of each attribute and level**

| **Dimensions** | **Attributes** | **Definitions/Explanation** |
| --- | --- | --- |
| Disease-related | Disease severity | The extent of the threat posed by the rare disease being treated or prevented to the patient’s health, and its impact on the patient’s daily life. This can specifically manifest as the disease’s effect on the patient’s survival time, quality of life, disability, or other functions. The degree of the disease impact includes the patient’s life year, quality of life, disability, or other functions. |
| Drug/treatment-related | Unmet needs | Reflects whether current medical resources, treatment methods, or drugs can effectively meet the needs for treating or preventing the disease. It is mainly assessed based on the existing treatment options in terms of prevention, cure, or improvement of the targeted disease’s condition. |
|  | Drug efficacy | Efficacy refers to the drug’s ability and extent to produce the expected therapeutic effect when treating the disease, that is, the clinical benefit brought to the patient by the drug at the individual level. |
|  | Improvement in health-related quality of life | Refers to the overall impact of the drug on the patient’s health through disease treatment, going beyond simple physiological efficacy. It focuses on whether the patient’s quality of life has significantly improved after treatment. It directly reflects the impact of treatment on the patient’s daily life quality and subjective well-being, including improvements in health-related quality of life (such as mobility, self-care, usual activities, pain/discomfort, or anxiety/depression) and other patient-perceived health (such as the impact on self-care ability and patient dignity). |
|  | Drug safety | Refers to the risks associated with the drug when used to treat the patient’s disease, including the potential side effects, toxic reactions, and the risk of adverse events. |
|  | Quality of drug evidence | Refers to the reliability, consistency, and sufficiency of scientific research data supporting the drug’s safety, efficacy, and other performance aspects. This includes the certainty, validity, relevance, and type of evidence. |
| Cost-related | Annual treatment cost per patient reimbursed by basic medical insurance | Refers to the portion of the annual treatment costs for each patient, which is paid or reimbursed by the basic medical insurance fund, within a given natural year. |

**Supplementary Table 1 Attributes and their definitions and explanations**

| **Attributes** | **Levels** | **Explanation** | **Basis for Setting Levels** |
| --- | --- | --- | --- |
| Disease severity | Low | Mild symptoms, low case fatality rate (CFR) (CFR < 10%, mortality < 100/100,000 people/year), minimal impact on quality of life | Based on current rare disease treatment status. |
|  | Moderate | More obvious symptoms, moderate CFR (CFR 10%–20%, mortality 100–200/100,000 people/year), may cause single disabilities or other factors affecting quality of life |  |
|  | High | Severe disease symptoms, high CFR (CFR > 20% or mortality > 200/100,000 people/year), or causes multiple disabilities |  |
| Unmet needs | Mature treatments available with good clinical outcomes | Certain medical solutions or relatively mature treatment pathways exist, with most patients achieving good clinical results. Overall, current medical conditions can meet most patients’ needs, though some areas may still need optimization, such as improving long-term prognosis, treatment convenience, or cost-effectiveness | Based on current rare disease treatment status. |
|  | Controllable treatments available to manage disease progression | Controllable treatments: aim to control disease progression, prevent or delay complications. Some interventions are available but their effects are limited, side effects are evident, or they are not suitable for all patient groups |  |
|  | No specific treatment available, only symptomatic/supportive treatment | Symptomatic/supportive treatments: aim to alleviate symptoms and improve the patient’s quality of life rather than treat the disease itself. Currently, there are few or no effective treatments, diagnostic tools, or support services available for the disease, and only symptomatic/supportive treatments to alleviate symptoms |  |
| Drug efficacy | Stabilizes disease | Treatment stabilizes the disease and prevents further deterioration | Based on current rare disease treatment status. |
|  | Partially improves or alleviates | Treatment can partially improve symptoms or disease state, but some symptoms or functional impairments still exist |  |
|  | Significantly improves or alleviates | Treatment can significantly improve symptoms or disease state, though it may not reach the standard of full cure |  |
| Improvement in health-related quality of life | No improvement in daily activity | After treatment, the patient still reports significant issues in multiple dimensions, such as mobility, self-care, usual activities, pain/discomfort, or anxiety/depression, with a low VAS score (e.g., 0-49). Daily life is still significantly affected. | Assumes that the patient’s health-related quality of life was poor prior to treatment, and improvement after treatment compared to before. Based on EQ-5D’s typical activity domains such as mobility, self-care (e.g., bathing, dressing), usual activities (e.g., work, study, or other daily tasks), pain/discomfort, anxiety/depression. |
|  | Partial improvement in daily activity | After treatment, the patient still reports some issues in the above dimensions, but they are not severe, with a moderate VAS score (e.g., 50-79). The patient can meet the basic demands of daily life. |  |
|  | Significant improvement in daily activity | After treatment, the patient reports only mild issues across the above dimensions, with a high VAS score (e.g., 80-100). The patient’s daily life is largely unaffected, with the ability to work, study, and socialize normally. |  |
| Drug safety | May cause severe adverse reactions | Severe reactions such as vomiting, rash, heart problems, or other symptoms that require medical intervention, significantly impacting daily life, leading to discontinuation of treatment, and possibly requiring hospitalization or other professional medical intervention | Based on the drug’s instructions and common classification of adverse reactions. |
|  | May cause moderate adverse reactions | Symptoms like persistent headaches, diarrhea, rash, or other symptoms that may affect daily life, requiring symptomatic treatment, typically controlled through simple medical intervention, and usually do not affect the continuation of medication |  |
|  | No or mild adverse reactions | Mild, reversible adverse reactions like nausea, headache, fatigue, with minimal impact on daily life |  |
| Quality of drug evidence | Low | The drug’s efficacy and safety are based on limited data, possibly from single small study, case reports, expert opinions, or retrospective analyses. This evidence is insufficient to fully prove the drug’s efficacy and safety, or there is significant uncertainty and controversy. | Based on the GRADE (Grading of Recommendations Assessment, Development and Evaluation) evidence grading system, the strength of evidence from different research designs, and the characteristics of orphan drugs. |
|  | Moderate | The drug’s efficacy and safety are based on a certain number of studies, including some non-randomized controlled trials, prospective cohort studies, or retrospective analyses. While these studies have limitations, they still provide some support. However, long-term data may be lacking or there may be discrepancies between studies. |  |
|  | High | Although rare disease research resources are limited, the drug’s efficacy and safety are supported by high-quality randomized controlled trials, systematic reviews, or meta-analyses. These studies have high internal and external validity, consistent results, and sufficient long-term follow-up data, with no significant controversies. |  |
| Annual treatment cost per patient reimbursed by basic medical insurance | 500,000 RMB | This level represents orphan drugs with high treatment costs, such as some gene therapies or biologics. For these drugs, the medical insurance fund pays a very high amount per patient. Based on a review of the current inpatient medical insurance reimbursement ceilings across China, the maximum reimbursement level may reach approximately 500,000 RMB. Therefore, 500,000 RMB is set as the threshold for basic medical insurance coverage of such drugs. | Based on the current annual treatment costs of drugs successfully included in national price negotiations and the cap line in various provinces in China. |
|  | 200,000 RMB | This level includes orphan drugs with relatively high treatment costs. Based on experience with national price-negotiations, most drugs with high treatment costs that are included in NRDL have annual treatment costs below 300,000 RMB. Therefore, the estimated reimbursement portion is around 200,000 RMB. |  |
|  | 80,000 RMB | This level includes orphan drugs with relatively low treatment costs. While still considered high-cost treatments, they are affordable for the basic medical insurance fund, such as those included in insurance after price reductions like Nusinersen. The overall annual cost is around 100,000 RMB, with the reimbursed cost being around 80,000 RMB or lower. |  |

**Supplementary Table 2 Levels and their explanations and rationale**
